# Eliminating air pollution disparities requires more than emission reduction

**DOI:** 10.1073/pnas.2505888122

**Published:** 2025-12-05

**Authors:** Libby H. Koolik, Robert D. Bullard, Esther Min, Rachel Morello-Frosch, Regan Patterson, Manuel Salgado, Nico Wedekind, Julian D. Marshall, Joshua S. Apte

**Affiliations:** ^a^Department of Civil and Environmental Engineering, University of California, Berkeley, CA 94720; ^b^Barbara Jordan-Mickey Leland School of Public Affairs, Texas Southern University, Houston, TX 77004; ^c^Front and Centered, Seattle, WA 98121; ^d^School of Public Health, University of California, Berkeley, CA 94720; ^e^Department of Environmental Science, Policy, and Management, University of California, Berkeley, CA 94720; ^f^Department of Civil and Environmental Engineering, University of California, Los Angeles, CA 90095; ^g^WE-ACT for Environmental Justice, New York, NY 10031; ^h^Department of Civil and Environmental Engineering, University of Washington, Seattle, WA 98195

**Keywords:** air pollution, environmental policy, environmental justice

## Abstract

In the United States, people of color are disproportionately and unjustly exposed to air pollution. Historically, environmental policy has emphasized aggregate emission reductions, yet major emission reduction scenarios do not sufficiently mitigate relative exposure disparities. Here, we show that without focusing on relative disparity (percent difference) in exposure, the only way to improve air quality and eliminate absolute exposure disparity is to eliminate all emissions (an unlikely outcome). We demonstrate that the relative disparity metric is ethically important and also a controllable societal feature that can reduce absolute disparity in exposure. We illustrate a range of approaches to air pollution policy that go beyond traditional emission reductions to meaningfully address exposure disparities. These strategies should be at the center of the future US environmental policy.

In the United States, people of color are disproportionately and unjustly exposed to higher-than-average concentrations of fine particulate matter (PM_2.5_), nitrogen dioxide (NO_2_), and several other harmful air pollutants (1–12). This inequality in exposure today is the product of more than a century of practices and policies, including racially discriminatory actions such as redlining, inequitable placement of emission sources, and more (6, 7, 13–17). For the past several decades, the Clean Air Act and other policies have reduced overall population-average exposures and *absolute* disparities in exposure, yet *relative* disparities in exposure between racial–ethnic groups persist (1, 3, 5, 18, 19). Recent research highlights that many emission reduction strategies, including business-as-usual (BAU) scenarios and scenarios with even greater emission reduction, surprisingly maintain the unjust status quo of racial inequality in air pollution exposure (18, 20–29). Here, we propose a conceptual framework to explain those findings and point toward scalable solutions to addressing these disparities.

Exposure disparities can be expressed as *relative* disparity (% difference) or *absolute* disparity (amount, e.g., μg/m^3^, ppb). Absolute exposure disparity is more relevant for health outcomes because increases in exposure to pollutants such as PM_2.5_ and NO_2_ are causally associated with adverse health outcomes (30, 31). *Relative* exposure disparity is a measure of the exposure inequality faced by a community and a fundamental indicator of environmental (in)justice. Here, we demonstrate how absolute disparity is strongly influenced by policy, land use, and planning choices that directly alter the relative disparity.

## Framework for Targeting Disparities

We propose a conceptual framework to understand how factors that are manipulable by policy and/or engineering can modulate air pollution disparities. This framework both explains the lack of progress in reducing relative exposure disparities and helps elucidate what makes a policy successful or unsuccessful at reducing or eliminating exposure disparities. For air pollution exposures, absolute disparity (*D_A_*) can be decomposed as the product of three straightforward and controllable components: emissions (*E*); population-average exposure factor ($\bar{\mathrm{XF}}$); and relative disparity (*D_R_*):

[1] $$D_{A}=E\times \bar{\mathrm{XF}}\times D_{R}.$$

Each of the four variables above represents an independent modulator of absolute exposure disparity. These terms can be independently estimated and then combined to quantify total impacts, decomposed from modeled or observationally constrained data to attribute contributions, or interpreted abstractly to assess how a policy intervention might influence each term. Each term is defined below:

- *E* (units: g/day) is the total amount of emissions contributing to exposures.
- $\bar{\mathrm{XF}}$ (units: μg/m^3^ exposure per g/day emitted) is the population-average exposure, per unit of emissions. This parameter depends on atmospheric processes (e.g., wind speed and direction, physical/chemical transformations) and source placement (average proximity of the source to people) and is related to the intake fraction, as defined in the literature (32–35).
- *D_R_* (units: decimal or %) is the percent difference in exposure for a subpopulation compared to the population average. This parameter indicates the spatial bias in pollution levels. In the equation, *D_R_* is represented in decimal form (e.g., 0.2); we report it in the text as a percentage (e.g., 20%).
- *D_A_* (units: μg/m^3^), the product of the three variables above, is the absolute difference in exposure for a subpopulation compared to the population average.

A crucial advantage of this framework is that this equation can be conceptual, or it can be applied mathematically to a specific emission source, geography, subpopulation, and pollutant (a mathematical derivation is included in *SI Appendix*). As an example, consider a policy aiming to reduce disparities in exposure to NO_x_ (oxides of nitrogen) from motor vehicles in a city. Here, we will demonstrate the value in applying this framework both conceptually and quantitatively.

Applying the framework conceptually first facilitates an understanding of how policy decisions drive changes in exposure disparity. Importantly, each component of the framework can be individually influenced by technology, policy, planning, and land use decisions. For motor vehicles, *E* is governed by economic and land-use decisions that affect activity (e.g., walkability, suburbanization, travel demand, relative costs, public transit options) and technology-based decisions that affect emission factors (e.g., diesel particulate filters or other emission controls, fuel economy, electrification). A policy that reduces *E* in-place without perturbing the spatial distribution of those emission sources is likely to only reduce absolute disparity by the same amount. $\bar{\mathrm{XF}}$ and *D_R_* both reflect land-use decisions (set mainly by local to national environmental, transportation, and urban planners), which impact proximity between people and pollution sources (e.g., roadway and highway networks). $\bar{\mathrm{XF}}$ reflects decisions that impact average proximity (e.g., whether sources are placed inside or outside of densely populated areas). Similar to the intake fraction, it facilitates comparisons of the relative *potential* to cause population-wide exposures. In contrast, *D_R_* reflects decisions that impact differences in proximity among groups; those reflect how segregated a city is (both for people and pollution sources), for example, whether roads were more likely to be placed in socioeconomically and politically marginalized communities. It is likely that a given policy could result in changes in multiple framework components. In such cases, Eq. **1** provides a useful conceptual model for considering how disparities might change. For example, a motor vehicle policy that reroutes trucks away from portside communities (↓ *D_R_*) but increases emissions (↑ E) due to higher vehicle miles traveled (VMT) might result in reduced absolute disparities so long as the compensatory increase in emissions is relatively low.

The framework can also be quantified for any proposed or implemented policy. Continuing with the NO_x_ example, a mobile source emission inventory (e.g., the Environmental Protection Agency’s [EPA] MOVES) could be used to estimate changes in NO_x_ emissions (*E*); a chemical-transport model (e.g., CMAQ Adjoint) could estimate unit-emission concentration sensitivities to changes in emissions ($\bar{\mathrm{XF}}$); and high resolution United States Census estimates could provide information about the demographically differential exposures (*D_R_*). Moving briefly away from the NO_x_ example, we note that the difficulty in quantifying framework variables for different policies will vary. For pollutants where the chemistry and transport are approximately linear (e.g., diesel particulate matter, black carbon), the analytical application is straightforward. Changes in framework components can be easily estimated for primary pollutants and certain individual secondary pollution pathways for which exposure can be linked directly back to the emission source. As is often the case for understanding air pollution, the application of the equation above is less straightforward for pollutants where secondary formation is not easily attributed to a single emitted precursor or is highly nonlinear (e.g., O_3_). One reason for this is that, as written, the equation disaggregates the average exposure factor for pollutants formed from secondary reactions of emissions of multiple precursor species. Another reason is that nonlinear chemistry can sometimes cause unintuitive relationships, including concentration increases when precursor emissions decrease. It is widely appreciated that O_3_ concentrations can increase in response to NO_x_ emission reductions in VOC-limited regimes (36, 37). In this case, a reduction in emissions (↓ *E*) could inadvertently increase population-wide exposures per unit emissions (↑$\bar{\mathrm{XF}}$). If a subpopulation experiences lower-than-average exposures as a result, that could be represented as a negative value for *D_R_*. In either case—be it a simple, approximately linear pollutant or a complex nonlinear pollutant—changes in the framework variables can be quantified using observationally constrained exposure estimates or atmospheric modeling tools. For direct policy attribution analysis, the level of model sophistication (i.e., full suite chemical-transport model for complex, nonlinear chemistry versus reduced-complexity or dispersion model for more simple pollutant) will vary depending on the specific behavior of the pollutant and geographic domain.

In fact, a key strength of the framework is the broad applicability across space, geographic scale, demographic group, and pollutant of interest. Moving beyond the NO_x_ example, the framework could also be used to reduce disparity in exposure to toxic metallic air pollutants from industrial sources across a given state, for example. Here, *E* is driven by economic activity, product supply and demand, and emission controls (e.g., baghouse filters for particulate metals). As with mobile sources, $\bar{\mathrm{XF}}$ and *D_R_* are driven by land use and zoning decisions. Regulators might target $\bar{\mathrm{XF}}$ alone by encouraging the shifting of industrial facilities away from urban areas and uninhabited areas. In comparison, *D_R_* could be influenced by permitting and regulatory decisions about where new sources can be sited. The process for locating emission sources can involve state-level regulators yielding “not in my backyard” (NIMBY) pressure from many communities, combined with greater political power and influence by wealthy, whiter communities. Avoiding statewide policies that are fully location-agnostic (e.g., cap-and-trade) may also help reduce *D_R_* from emission sources at the statewide level. A similar exercise could be performed for other combinations (e.g., nationwide emissions of black carbon from diesel rail engines, or PM_2.5_ emissions from construction equipment in an airshed). Considering the application of the framework across various spatial scales, the key is defining the relevant and appropriate system boundary (e.g., city, region, state). The system boundary will determine the total amount of *E* and provide the population basis from which $\bar{\mathrm{XF}}$ and *D_R_* are estimated. For a given geographic domain, the population group of interest may change. Similarly, the functional unit of consideration (e.g., census block, county) may change as a function of the total geographic extent.

Another potential application of the framework could combine *E* and $\bar{\mathrm{XF}}$ into one concentration term (*C*) that represents the average population-wide exposure. This term could be derived using atmospheric models or spatially complete, observationally constrained data. A benefit of this approach is that it avoids simplifying complex chemistry or meteorological processes. However, this approach substantially limits the utility of the framework for ideating potential control strategies: Without including total emissions and spatial proximity as independent, direct levers of control when applying the framework conceptually, it is less obvious which policies reduce emissions in-place versus meaningfully redistribute the pollution burden.

The disparity metrics correspond to specific subpopulations (e.g., by race, income, etc.) or locations [e.g., by disadvantaged community (DAC) designation, using spatial mapping tools]; selecting which subpopulation to investigate is a critical consideration when applying the framework above. In general, exposure disparities by race/ethnicity in the United States are larger than, and statistically distinct from, disparities by income (4, 5, 7, 10–12, 18). Multiple tools and metrics have been used for identifying priority locations for emission mitigation (38–40). One example is the Biden Administration’s Climate and Economic Justice Screening Tool (CEJST), developed as part of the Justice40 initiative (38). Examples of state-level tools include the California Communities Environmental Health Screening Tool (CalEnviroScreen) and the Washington State Environmental Health Disparities map; these use similar methods as CEJST, but with different sets of indicators for pollution burden, sociodemographic factors, and health outcome sensitivity variables (39, 40). For applicability to all federal agencies, CEJST also includes indicators for climate risks, transportation, workforce development, and housing. Notably, because of legal restrictions and the perceived potential for legal challenges to the explicit inclusion of race and ethnicity in policymaking in the United States, these tools, which often guide decisions about the allocation of funding, tend to exclude race/ethnicity. Typically, they instead use a combination of socioeconomic (e.g., median household income), environmental (e.g., projected flood risk), and health (e.g., childhood asthma rates) indicators to identify overburdened areas. While these screening tools provide a useful, data-driven approach to identifying overburdened communities, if they exclude race/ethnicity, emission reduction efforts employing those definitions may not reduce disparities by race/ethnicity (24).

Trends in other countries may differ. The framework can usefully be applied in other countries or contexts, reflecting aspects of population exposure disparities that are relevant there. Additionally, while we focus here on individual demographic specifications (e.g., race/ethnicity), intersectionality should be carefully considered when determining the most relevant sub-population for a specific application of the framework.

## Policy Application

**Fig. 1.**
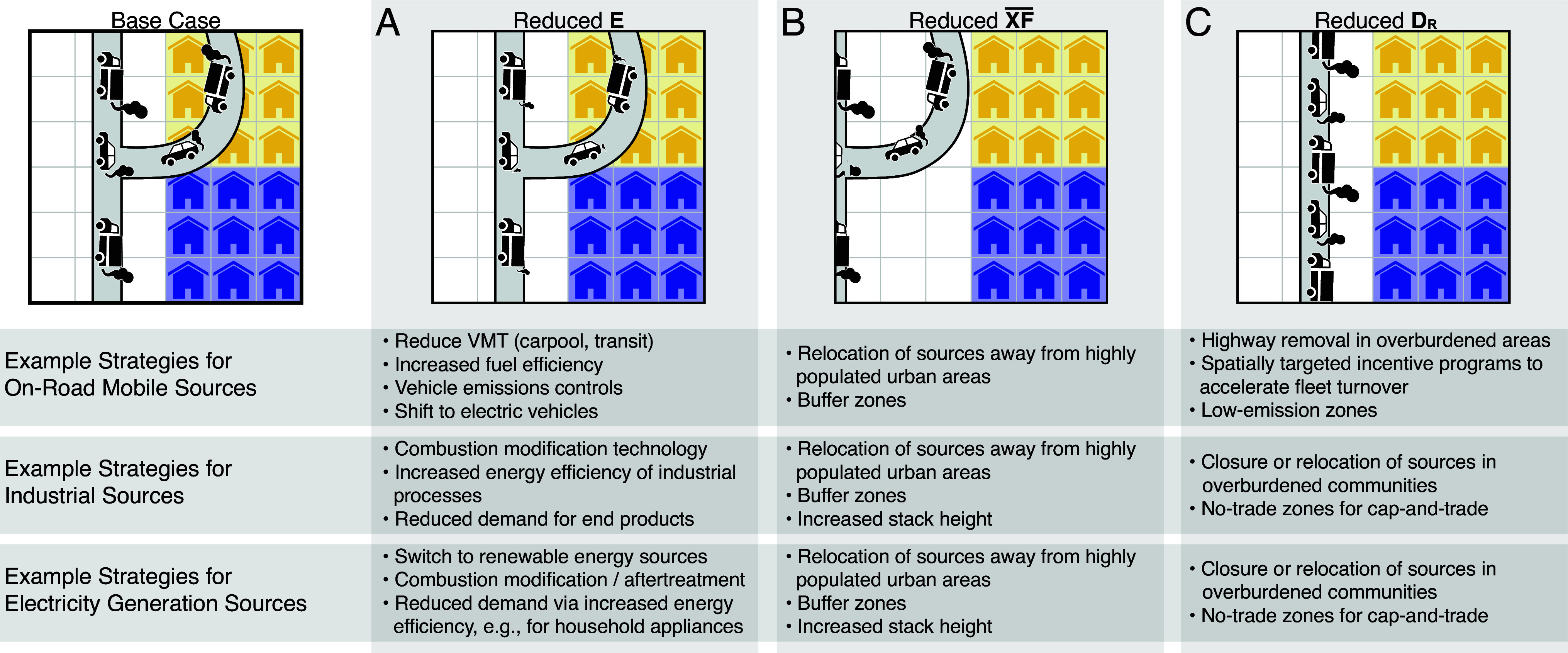
A partial solution set for policies that meaningfully alter each framework component. Here, we give example policies that would target each framework variable. Example strategies are shown for on-road mobile sources, industrial sources, and electricity generation sources. In the Base Case, the cartoon depicts a major highway located near a residential area and running through the community of yellow houses (overburdened group). Subsequent columns show changes that primarily target one of the framework components. (*A*) Emissions are reduced from vehicle tailpipes. (*B*) The highway is moved away from all people but is still spatially biased. (*C*) The highway through an overburdened community is removed and traffic is redistributed elsewhere (i.e., to non-spatially-biased roads). In practice, many strategies will affect more than one component. For example, spatially targeted emission reductions of drayage trucks driving through an overburdened community would likely reduce all four components; the spatial targeting is needed to change the relative disparity.

Example policies, and their potential effect on the terms in the equation above, are given in Fig. 1. The illustrative strategies in Fig. 1 support longstanding arguments made by environmental justice advocates about the need to target relative disparity in environmental decision-making.

In general, there are two categories of policies that can target relative disparity in exposure. The first is the decommissioning or relocation of specific sources away from overburdened communities. The second makes targeted investments in overburdened communities, to either reduce current emission or avert BAU emission growth. Whereas strategies that reduce overall society-wide emissions (e.g., emission control technologies for all sources) can reduce exposures and absolute disparities, only those policies that modify the spatial distribution of emissions (e.g., by prioritizing emission control in overburdened communities) will reduce relative disparities. Thus, reductions in relative disparity do not arise from spatially uniform reductions in emissions.

It is useful to consider *who* sets and manages policies relevant to each term in the framework. Policies that target changes in *E* are likely to be set by air pollution regulatory agencies (e.g., EPA, state EPAs, local and regional air boards) strengthening emission control requirements. Changes to $\bar{\mathrm{XF}}$ and *D_R_* are more likely governed by combinations of regulatory agencies. For example, local planners can decide where new projects, facilities, and housing developments can or cannot be sited; transportation agencies can decide where to build or widen roads, or whether to invest in transit infrastructure to shift travel modes away from car-dependency; and local and state environmental regulators can decide where to invest in local community-level infrastructure (e.g., bus electrification, exhaust stack retrofitting, electric vehicle charging infrastructure). Ultimately, these factors should be considered in the context of existing zoning laws, land use specifications, and land suitability as well as the processes by which these land use parameters change.

Consider again the NO_x_ scenario above. To reduce relative disparities, policies should prioritize (i.e., spatially target) reducing the emissions that most impact overburdened communities. For example, current efforts in logistics hubs around the United States emphasize electrification of short-haul drayage trucks; that step often would disproportionately benefit overburdened communities. Other examples of location-based policies for vehicles that could address relative disparities include freeway rerouting or removal to reduce disparate impact on overburdened communities (41), low-emission zones (42), or targeted incentive programs to accelerate the replacement of older cars in low-income communities (43). While such policies exist, there is not robust evidence that they are yet at sufficient scope or scale to meaningfully reduce relative exposure disparities (18). For such location-based emission control policies to successfully reduce relative disparities, emission reductions in overburdened communities must *meaningfully outpace the aggregate economy-wide emission reduction rate, on a sustained basis*. In our experience, the equity and overall benefit of this strategy is not yet widely appreciated in mainstream policy circles.

## Combine Approaches to Reduce Disparity

Next, we explore the utility of the framework above (Eq. **1**) and the benefits of simultaneously reducing all three components (*E*, $\bar{\mathrm{XF}}$, and *D_R_*), via two approaches: i) by developing and applying a simplified model that illustrates key findings and offers new insights into how to reduce disparities, and ii) by using the equation to help contextualize, and add a new dimension to, previously published research. The equation helps frame an important problem (i.e., how air pollution policies can structure emission reductions so as to reduce or eliminate disparities) and calls out a new approach to its resolution.

In the simplified model, population density decreases radially outward from the city center, with west-to-east segregation between two demographic groups. Pollution levels exhibit a spatial bias that reflects i) inequitably sited emission sources and ii) a Gaussian decay of concentrations moving away from the emission source. In *SI Appendix,* Fig. S1, we employ the illustrative model to demonstrate that each framework component (*E*, $\bar{\mathrm{XF}},$ and *D_R_*) can be independently targeted. Results here consider a primary, conserved pollutant; in the SI we provide results for multiple example evaluations of a reactive primary pollutant.

**Fig. 2.**
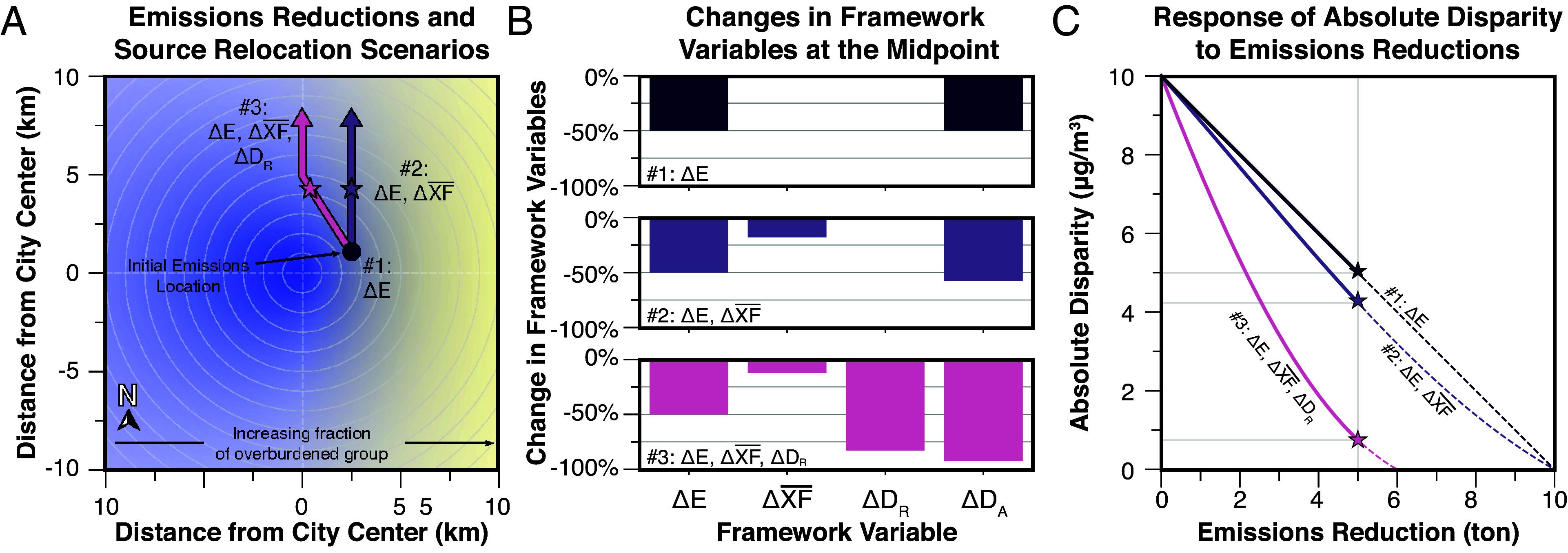
Absolute disparity is mitigated faster when policy actions incorporate more framework components. To illustrate, we modeled pollution levels and exposure disparities for three scenarios in a segregated hypothetical city. (*A*) A map of the city, showing the scenarios and the two demographic groups (blue, yellow). Each scenario includes a constant rate of emission reduction. In scenario 1, the spatial distribution of relative concentrations is unchanged, with the peak concentration at the black dot. (Concentrations decrease as the emissions decline, but relative spatial patterns are unchanged.) In scenarios 2 and 3, emissions are relocated over time, moving along the arrows as emissions are reduced: reducing the average exposure factor but maintaining the inequality (scenario 2) or eliminating inequality and reducing average exposure factor (scenario 3). (*B*) Changes for each parameter, for the three scenarios, for a 50% reduction in emissions (indicated by a star). The change in relative disparity shown is the percent change in the decimal version between the start and the point at which 50% of emissions are reduced (e.g., 0.1 to 0.12 is a 20% reduction). (*C*) Absolute disparity as emissions are reduced, from 0 to 100%, for the three scenarios. This figure illustrates that, for a given level of emission reduction, policies that adjust the *pollution location* to reduce spatial bias can achieve a much larger reduction in (or can eliminate) relative disparity; adjustment in pollutant location can arise from relocation of sources and/or from greater rates of emission reduction in overburdened communities.

We consider how concentration disparities respond to changes in the magnitude and location of emissions following three scenarios in which emissions are reduced over time (Fig. 2).

- Scenario 1. The spatial bias in pollution levels remains unchanged.
- Scenario 2. Emissions are spatially shifted in a way that reduces the *average* exposure factor but not the *relative* disparity in exposure (e.g., away from the city center but maintaining the spatial bias).
- Scenario 3. Emissions are spatially shifted in a way that reduces the average exposure and the relative disparity in exposure.

The first change reduces *E*; the second, *E* and $\bar{\mathrm{XF}}$; the third, *E*, $\bar{\mathrm{XF}}$, and *D_R_*. These three approaches lead to increasingly more impactful changes on absolute disparity (Fig. 2 *B* and *C*). At the midpoint of the emission reduction pathways, the equivalent emission reduction yields greater reductions in absolute disparity when complementary actions also reduce the average exposure factor and relative disparity. The reciprocal finding from this figure also holds: the same reduction in absolute disparity can be achieved more rapidly (i.e., with ~60% less reduction in emissions) if average exposure factor and relative disparity are also reduced (see also *SI Appendix,* Fig. S2). However, given that additional emission reductions lead to improved health overall, we highlight here the case where equivalent emission reductions result in increased equity benefits, rather than the case where equivalent equity benefits are achieved with differential emission reductions. An additional policy scenario is modeled in *SI Appendix,* Fig. S3; in *SI Appendix,* Fig. S4, we add a hypothetical first-order rate decay to our concentration gradient to demonstrate our conclusion’s robustness to the simplicity of this illustrative model for describing exposure patterns for a shorter-lived pollutant.

Several key insights emerge from Fig. 2 regarding modern environmental regulations. First, relative disparity is an independently manipulable input to the equation that can be explicitly targeted by policies, and not necessarily a natural outcome of untargeted emission reductions. Future policy efforts should be designed to intentionally address relative disparity wherever possible, and quantitatively track progress over time toward reducing these disparities. When more than one framework variable is reduced, the reduction in absolute disparity in exposure occurs much more rapidly. In fact, the pathway to reducing absolute exposure disparity the fastest requires the engagement of all the framework components. The only case where absolute disparity is fully mitigated without the complete elimination of emissions occurs when relative disparity is also reduced. Widespread technological interventions are important but are only part of the solution for mitigating exposure disparities.

An advantage of the simple model is that it can highlight underlying mechanisms and easily track exposures and disparities without being obscured by methodological details and the complexity, noise, and uncertainties present for large models. However, it lacks important features of true atmospheric physics and chemistry (e.g., chemical transformation, meteorology, deposition).

**Fig. 3.**
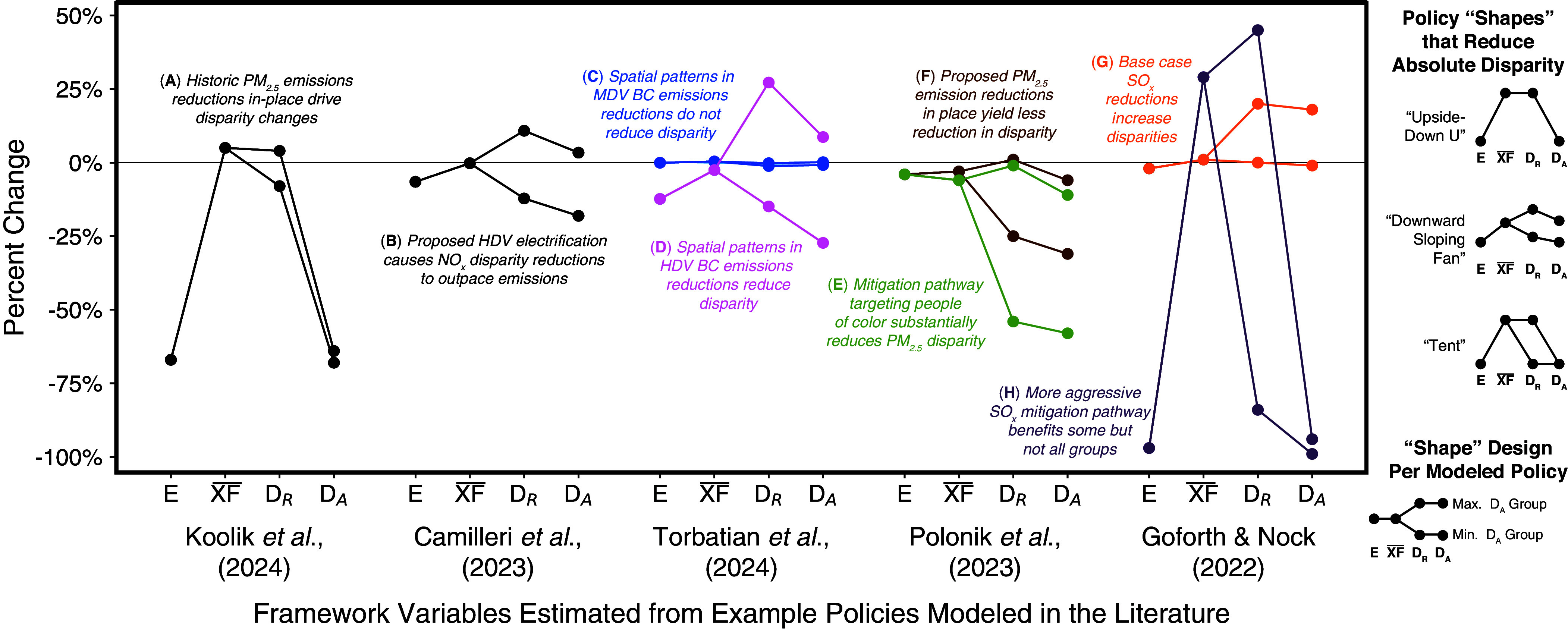
The conceptual framework highlights insights from policy evaluations in five peer-reviewed modeling studies taken from the literature, representing a range of modeling approaches and pollutants. The modeling results are decomposed into changes in emissions (*E*), average exposure factor ($\bar{\mathrm{XF}}$), relative disparity (*D*_*R*_, in decimal form), and absolute disparity (*D*_*A*_) for two representative groups. The patterns highlight mechanisms of the changes in exposure disparity. (*A*) Koolik et al. (18) and (*B*) Camilleri et al. (20, 30), highlight two modes of policy design using a reduced-complexity model and a comprehensive chemical transport model, respectively. While the “upside-down u” shape reflects changes in absolute disparity being primarily driven by changes in emissions in Koolik et al. (18), the “downward sloping fan” in Camilleri et al. (20, 30) highlights how a given emission reduction can yield disproportionately larger reductions in absolute disparity if relative disparity is also reduced. The “tent” shape [Goforth and Nock (23); line H] represents a policy that changes relative disparity, but its impact on absolute disparity is outsized by a large reduction in emissions. In the latter three papers (lines *C* - *H*), example policies demonstrate the range of findings across two modeled scenarios. A common theme of the policies that are more successful in reducing absolute disparity is attention to source patterns that disproportionately affect the most disparately exposed group. Additional evaluations with these datasets and details about the policies modeled are included as *SI Appendix*, Fig. S5 and Table S1. For visual simplicity, for each set of policy evaluations, the figure displays only the groups with the maximum and minimum change in absolute disparity; a full display of all policies evaluated in those prior studies is given in *SI Appendix,* Fig. S5.

Accordingly, we next apply the framework to existing modeling from the literature. Specifically, we consider eight example policy scenarios from five recent analyses; those analyses involve comprehensive modeling tools (CMAQ, Polair3D, InMAP), and several spatial scales (United States, California, Chicago region, Toronto region) and a diverse array of primary and secondary pollutants (primary PM_2.5_, black carbon [BC], NO_2_, and secondary inorganic aerosol resulting from NO_x_ and SO_x_ emissions). The framework given here enables us to extract high-level insights from these literature results to pinpoint the mechanisms driving whether or not a policy reduces absolute exposure disparities (Fig. 3). (Additional examples, beyond the policy scenarios and pollutants considered here, are in *SI Appendix*, Table S1 and Fig. S5.)

From these eight scenarios, as represented using the equation above, a few archetypal patterns emerge (Fig. 3). The first is an “upside-down u” shape, as seen in the Koolik et al. (18) results. Here, absolute disparities decline due to emission reductions in the absence of meaningful changes in spatial patterns (analogous to scenario 1 in Fig. 2). Second, a “downward sloping fan” [for example, the Camilleri et al. (20) truck electrification] reflects a scenario where emissions and relative disparity both decrease. In Fig. 3, only the pathway that specifically targets exposure disparities for people of color [the green curve from Polonik et al. (21)] decreases all three variables, suggesting a more complex analog to scenario 3 in Fig. 2. A common theme amongst the policies in this second category was an explicit targeting of sources that disparately expose a specific demographic group. The final pattern is a “tent” shape, which occurs in the very aggressive carbon cap pathway from the Goforth and Nock (23). This scenario involves the relative disparity for one group increasing, but this is counteracted by such substantial emission reduction that the absolute disparity reduced for that group, too. No policies were identified in our reanalysis that substantially changed $\bar{\mathrm{XF}}$.

A central theme emerges from this set of policies we assessed. In general, emission changes (*E*) are the strongest driver of changes in absolute disparity. Indeed, the only policies where reductions in absolute disparity outpaced emission reductions are those that combine emission reductions with spatial redistribution. Specifically, these policies redistribute emissions in ways that reduce the systemic bias in how emitting sources are distributed across different areas (*D_R_*). Consider the example from Koolik et al. (18) (Fig. 3*A*), which looked at the historical trends in California’s climate and health-oriented on-road mobile source policy. Here, the “upside-down u” reflects decades of technological innovation (e.g., increased fuel efficiency, exhaust control technology) but minimal spatial redistribution of vehicle activity. As a result, the change in absolute disparity is nearly equal to the change in emissions. Compare this case with the “People of Color” pathway from Polonik et al. (21) (Fig. 3*E*). Here, there is an additional reduction in absolute disparity for both groups modeled, resulting from the synergy of simultaneous reductions in $\bar{\mathrm{XF}}$ and *D_R_*. These benefits arise because the emission reduction pathway was designed to target emission reductions in communities of color, which is a direct intervention in the spatial bias of pollution sources. At first glance, reducing emissions in-place may seem more feasible. For example, reducing the average motor vehicle emission rate as the vehicle fleet naturally turns over could be easier than relocating a highway. While possible, highway relocation is likely to be met with NIMBY pressure and high infrastructure costs. In contrast, spatially targeted emission reductions and infrastructure investment may have fewer political barriers. Programs that bring low-emission technology (e.g., electric school buses), sustainable investments (e.g., land conservancies), and/or health-protective interventions (e.g., filter distribution centers) to overburdened communities are examples of spatially targeted, practicable, and impactful policies that are happening in various places across the United States.

## Call-to-Action for Policy

Evaluations of exposure equity outcomes of historical and proposed policies tend to treat relative exposure disparity as a policy outcome, rather than a modifiable policy input. Multiple recent publications highlight that historic and future policies tend to reduce average exposures and absolute disparities, but not relative disparities. That outcome highlights the urgent need to rethink how we understand, quantify, and eliminate air pollution exposure disparities. While there has been substantial fluctuation about the extent to which federal agencies in the United States will be able to pursue environmental justice (e.g., Trump administration elimination of Environmental Justice offices in the EPA) and the existence of deference to federal agencies (e.g., overturning *Chevron* doctrine via *Loper Bright Enterprises v. Raimondo*) (44), this does not diminish the central findings or message of this work. A central insight of this body of work is that many of the factors that drive relative disparity come down to the spatial configuration of emission sources in the places people live. Many of these decisions are ultimately controlled by regional, state, and local planning. Without a unifying federal directive to pursue these goals, regional and local legislation must be more prescriptive and explicitly target the elimination of population-based disparities in exposure. This will require a system of tracking and mitigating relative disparity in exposure across racial/ethnic, class, and other demographic lines. The framework proposed here provides a range of complementary approaches to air pollution policy to meaningfully address exposure disparities.

To this end, we recommend the immediate implementation of the following two actions. First, state, regional, and federal policymakers should adopt enforceable standards targeting air pollution exposure disparities (both *D*_*A*_ and *D*_*R*_). The standards set for *D*_*A*_ and *D*_*R*_ should be tracked, monitored, and benchmarked as part of air pollution regulatory compliance, consistent with regulatory tracking of emissions (e.g., National Emissions Inventory, Toxics Release Inventory) and concentrations (e.g., ambient air quality monitoring network). Our framework provides a set of metrics that could support this ongoing evaluation by public health agencies. This data tracking would be in coordination with existing efforts to quantify temporal trends in population demographics (e.g., United States Census Bureau estimates), pollution source siting (e.g., emission inventories), and ambient air pollution (e.g., regulatory monitoring network).

Second, policymakers should initiate policy design by identifying feasible opportunities to mitigate *D*_*R*_ by targeting the underlying race-based and other discriminatory causes of the persistent exposure inequities rather than focusing on bulk emission reductions. Policies that meaningfully reduce relative disparity in exposure (*D*_*R*_) generally reduce or eliminate emissions in locations such that they directly reduce the disproportionate pollution burdens caused by the legacy of racially discriminatory policies. Without the explicit consideration of legacies of racial discrimination in designing solutions, we cannot expect to dismantle persistent disparities in exposure. While recent Supreme Court case law has upended well-established equal protection law with its decision in *SFFA vs. Harvard* and *SFFA vs. UNC*, these cases have not overturned legal standards for compliance with federal civil rights in other areas (45). As such, regulatory agencies still have an obligation to evaluate their policies and practices to ensure that they address and at minimum do not undermine equal protections based on race or otherwise.

Environmental justice advocates have focused regulatory attention toward the evaluation and amelioration of *cumulative burdens*, as many overburdened communities are disproportionately exposed to emissions from many independently-regulated source categories (4, 9, 46). The aggregate effect of this pollution is important, as health outcomes are associated with an individual’s total pollution exposure, and thus the health disparities are associated with the disparity in total exposure (31, 47, 48). While our framework focuses here on individual source sectors, its future application will need to address the mitigation of cumulative burdens.

Historically, air pollution policy has focused on tracking and reducing aggregate emissions and exposures. Here, we show that to mitigate absolute disparities in exposure to harmful air contaminants, regulators must apply controls to not only emissions but also to the relative inequality in the location of the disproportionate burdens. We have provided a conceptual and mathematical framework for considering and quantifying how actions and policies can influence key attributes (e.g., emissions, disparities), at the local, state, or national level. The current regulatory landscape challenges decision-makers to enact legislation that specifically addresses racial and other population-based exposure disparities. Our framework provides a starting point and clear pathway for expediting these much-needed reductions in relative exposure disparity.

## Methods

To demonstrate the influence of each of the framework components on the absolute disparity in exposure, we devised an illustrative model of a simple system. The illustrative model allows us to control for very specific variables within a hypothetical system boundary. For simplicity, this model system has only two population groups (*α* and *β*) and one emission release point. This example is meant to be illustrative; for a full understanding of a population group’s exposure, care should be taken to consider an individual’s full range of microenvironmental exposures. A complete treatment might consider the full spatiotemporal variation of how diverse populations move around in geographic microenvironments including indoor and outdoor settings. In practice, these issues are generally neglected in the literature because they are difficult to measure and quantify (49, 50).

We start the illustrative model by creating a square two-dimensional grid (200 × 200) that assigns the population of two groups along a linear population distribution, such that two equal groups are created with a higher density of Population *α* on the right-hand side of the grid. Additionally, to approximate the distribution of overall residents in an urban area, the total population density is the highest in the city center and decays radially outward. An emitting source is placed within the grid at a specific location (*x_0_*, *y_0_*) with an emission rate (*E*). This emitting source represents either a single emission point or the population-weighted mean emission location. Consistent with the Gaussian plume approach used in standard air pollution dispersion modeling, concentrations are estimated across the grid using a two-dimensional Gaussian distribution centered at the emission release location:

[2] $$C\left(x,y\right)=\frac{E}{\mathrm{nuH}}\times exp\left(-\left(\frac{{\left(x-x_{0}\right)}^{2}}{2\sigma_{x}^{2}}+\frac{{\left(y-y_{0}\right)}^{2}}{2\sigma_{y}^{2}}\right)\right),$$

where *n* is the number of grid cells (*n* = 200), *u* is the average wind speed (5 m/s), and *H* is the average boundary height (100 m). For simplicity, the SD in the *x*- and *y*- directions (*σ_x_* and *σ_y_*) are both assumed to be the same. For each run of the illustrative model, population-weighted mean exposures (PWM_α_ for group *α*; PWM_T_ for total population) and absolute disparities in exposure (D_A,α_) for Population *α* are calculated using Eqs. **3** and **4**, respectively.

[3] $${\mathrm{PWM}}_{\alpha }=\frac{\sum_{i=1}^{n}\left(P_{i,\alpha }\times C_{i}\right)}{\sum_{i=1}^{n}P_{i,\alpha }},$$

[4] $$D_{A,\alpha }={\mathrm{PWM}}_{\alpha }-{\mathrm{PWM}}_{T}.$$

For Fig. 2, the illustrative model is used to estimate the combined impacts of reducing total emissions, the average exposure factor, and the relative disparity for Population *α* on the absolute disparity for Population *α* from a given emission source category. To do this, the model is run a thousand times for each of three illustrative scenarios. In all three scenarios, the total emissions are reduced from 10 g/s to approximately 0 g/s over a thousand linear steps. For the first scenario (Decrease *E* Only), all other variables are constant. In the second scenario (Decrease *E* and $\bar{\mathrm{XF}}$), the average location of the emission source is moved at each model step in a perpendicular direction to the linear population distribution. This simulates a policy that mainly affects the average population proximity to an emission source and not the relative inequality in source location. Finally, the third scenario (Decrease *E*, $\bar{\mathrm{XF}}$, and *D_R_*) moves the average source location at an angle away from the overburdened group and the full population at each model step until relative disparities are eliminated, simulating a policy that also reduces the relative inequality in source placement. After reaching a more equitable location in the east-west direction, the third scenario continues to move the source away from the highly populated city center, thereby further reducing the average population exposure via $\bar{\mathrm{XF}}$.

Additional applications of the illustrative model are included as *SI Appendix,* Figs. S2 and S3. All key parameters of the model in these figures are the same as described for Fig. 2; however, the mitigation pathways vary as illustrated. In *SI Appendix,* Fig. S4, we add first-order rate loss to the illustrative model to test the model’s robustness to an additional process. In this version of the illustrative model, the concentration is first calculated using Eq. **2** and then updated using Eq. **5**:

[5] $$C\left(x,y\right)=C\left(x,y\right)\times exp\left(-\frac{\mathrm{kd}}{u}\right),$$

where *k* is a first-order rate constant that substantially competes with the dilution over the domain, and *d* is the distance from the source (units: m). Two transects of the concentration with and without this first-order rate loss are included as *SI Appendix*, Fig. S4 *F* and *G*.

## Supplementary Material

Appendix 01 (PDF)

## Data Availability

Code files used to generate the illustrative model figures have been deposited in Zenodo (51). To access the data used in the analysis for Fig. 3, please refer to the original publications (18, 20–23, 25).
