# Supplementary material for: Eliminating air pollution disparities requires more than emission reduction
Source: Proc Natl Acad Sci U S A. 2025 Dec 5;122(50):e2505888122. doi: 10.1073/pnas.2505888122 (PMC12718300; doi:10.1073/pnas.2505888122)
Supplement: Supplementary file 1 — Appendix 01 (PDF) [file pnas.2505888122.sapp.pdf]

PNAS

Supporting Information for

Eliminating air pollution disparities requires more than emission reduction

Libby H. Koolik, Robert D. Bullard, Esther Min, Rachel Morello-Frosch, Regan Patterson, Manuel Salgado, Nico Wedekind, Julian D. Marshall, Joshua S. Apte*

*Corresponding author: Joshua S. Apte
Email: apte@berkeley.edu

This PDF file includes:

Supporting text
Figures S1 to S5
Table S1
SI References

Supporting Information Text

Mathematical Derivation of the Framework.

Consider a system that has a number of population groups (*k* = {*a*, *b*, *c*, …}) exposed to emissions from a variety of sources. The system has total emissions (*E*) resulting in a distribution of concentration throughout *n* grid cells. We define each group's exposure as a population-weighted mean exposure (PWM_k):

$$PWM_k = \frac{\sum_{i=1}^{n}(P_{i,k} \times C_i)}{\sum_{i=1}^{n} P_{i,k}} \qquad \text{(Eq. S1)}$$

where $P_{i,k}$ is the population of group *k* in grid cell *i* and C_i is the concentration of the pollutant in grid cell *i*. We define two metrics for estimating exposure disparity. We calculate the absolute disparity ($D_{A,k}$) as the difference between an individual group's exposure (PWM_k) and the total population exposure (PWM_T):

$$D_{A,k} = PWM_k - PWM_T \qquad \text{(Eq. S2)}$$

The relative disparity ($D_{R,k}$) is the percentage difference between an individual group's exposure and the population exposure (Eq. S3).

$$D_{R,k} = \frac{PWM_k - PWM_T}{PWM_T} \qquad \text{(Eq. S3)}$$

We propose the following mathematical framework:

$$D_{A,k} = E \times \overline{XF} \times D_R \qquad \text{(Eq. S4)}$$

The exposure factor ($\overline{XF}$) relates the total population-wide exposure to the total system-wide emissions and is defined below in Eq. S5.

$$\overline{XF} \equiv \frac{PWM_T}{E} \qquad \text{(Eq. S5)}$$

If we substitute Eq. S3 and Eq. S5 into the right-hand side of Eq. S5, we are left with the following equation:

$$D_{A,k} = E \times \frac{PWM_T}{E} \times \frac{PWM_k - PWM_T}{PWM_T} \qquad \text{(Eq. S6)}$$

Similarly, we can substitute Eq. S2 into the left hand side of Eq. S6:

$$PWM_k - PWM_T = E \times \frac{PWM_T}{E} \times \frac{PWM_k - PWM_T}{PWM_T} \qquad \text{(Eq. S7)}$$

Canceling terms on the right-hand side of Eq. S7 demonstrates equivalence:

$$PWM_k - PWM_T = PWM_k - PWM_T \qquad \text{(Eq. S8)}$$

It is also useful to contextualize these terms with an example of a real-world policy application to show how the units properly cancel. Consider the following units for Eq. S4 given exposure to $PM_{2.5}$ (exposure units: μg/m^3) from on-road vehicles.

$$\left[\frac{\mu g\ PM_{2.5}}{m^3}\right] = \left[\frac{g\ PM_{2.5}}{year}\right] \times \left[\frac{(\frac{\mu g\ PM_{2.5}}{m^3})}{(\frac{g\ PM_{2.5}}{year})}\right] \times [\%] \qquad \text{(Eq. S9)}$$

The framework here builds on a previously published population-exposure framework, intake fraction (*iF*), as defined in the literature (1–4). Specifically, $\overline{XF}$ relates to *iF* as follows:

$$\overline{XF} = \frac{iF}{P \times Q_B} \qquad \text{(Eq. S10)}$$

where *P* is the exposed population and Q_B is the population-average breathing rate (m^3 $person^{-1}$ y^{-1}). Intake fraction generally incorporates population-averages; it (like $\overline{XF}$) accounts for, but does not shed light on, exposure disparities.

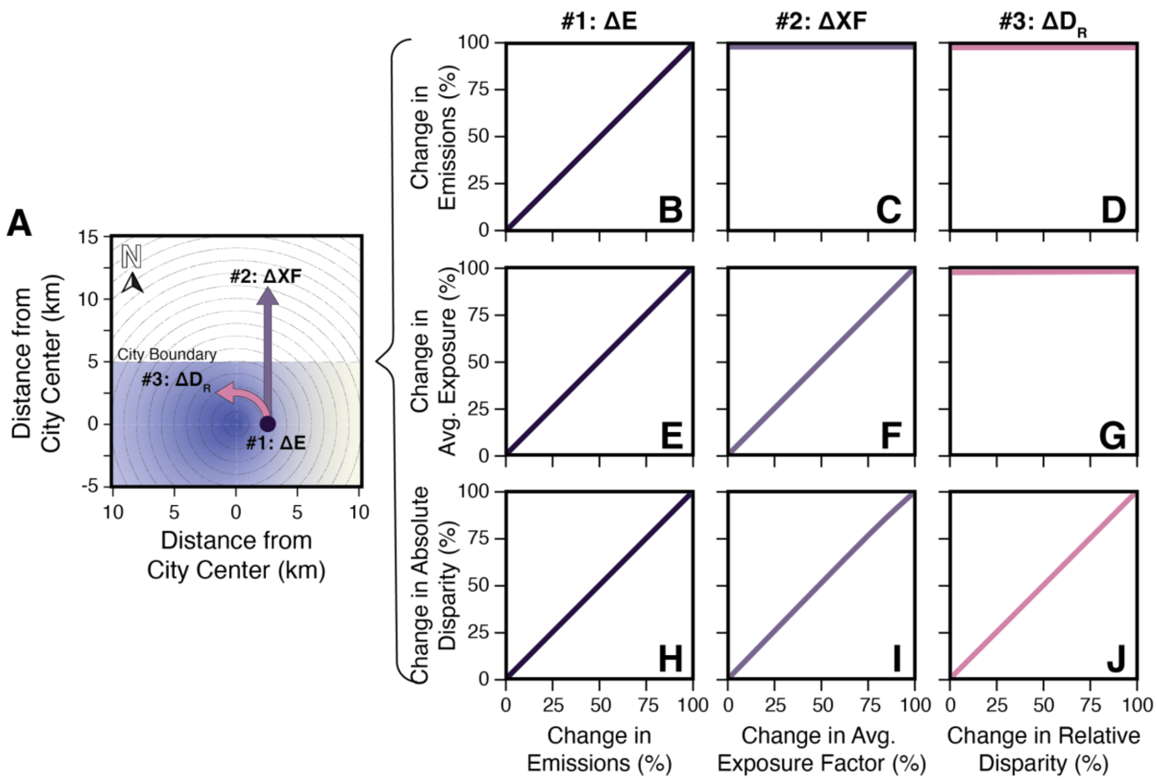

Fig. S1. Impacts of individual framework variables can be isolated in the illustrative model. Here, we demonstrate how each of the framework components is an independent variable of the total system-wide emissions, population-average exposure, and the absolute disparity. We also confirm the multiplicative relationships between the framework variables and output variables. As suggested by the framework, mitigating only emissions results in reduction in all three output variables, whereas mitigating the only relative disparity reduces only the absolute disparity. The three model scenarios are depicted in (**A**). In the first scenario (black), emissions are reduced in place (i.e., only emissions reduce). In the second scenario (purple), emissions are held constant as the source is moved outside of the urban boundary. In this scenario, the emissions are still closer on-average to the overburdened group, even as the source moves away from all residents of the city (i.e., only the average exposure factor reduces). In the third scenario (pink), emissions are held constant as the source moves away from the overburdened group but along a line of constant population density (i.e., only relative disparity reduces). In (**B**) through (**J**), the normalized modeled results are presented in a quasi-tabular format. The first row shows the total emissions (**B**-**D**), the second row is the population-average exposure (**E**-**G**), and the third row is the absolute disparity (**H**-**J**). The columns highlight the variable being changed in each scenario: the left-most (**B**, **E**, **H**) changes only emissions, the center (**C**, **F**, **I**) changes only the average exposure factor, and the rightmost (**D**, **G**, **J**) changes only the relative disparity. For simplicity in presentation, each variable is normalized relative to the initial condition (i.e., full emissions at the starting point).

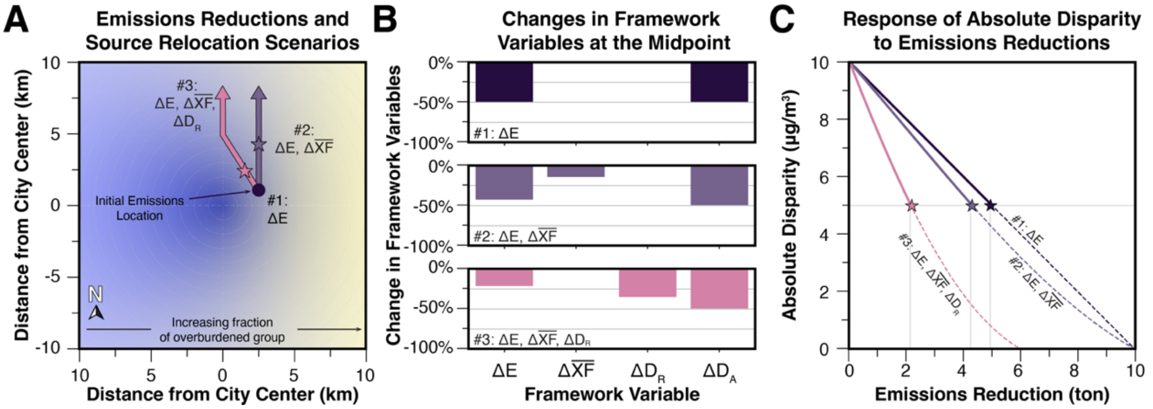

Fig. S2. Absolute disparity is mitigated with lower emission reductions when more framework components are incorporated into theoretical policies. The same three scenarios modeled in Fig. 2 are compared at the point at which all three achieve 50% reduction in absolute disparity. (**A**) Three theoretical emission reduction scenarios are depicted atop a simplified city. In Scenario #1, emissions are reduced in-place; in Scenarios #2 and #3, the average source location moves along the arrow as emissions are reduced. The stars represent the point at which the absolute disparity has reduced by approximately 50% in each curve; the influence of changes from each framework variable at this point are depicted in (**B**). (**C**) The absolute disparity in exposure for members of the overburdened group is calculated for each policy at each model step.

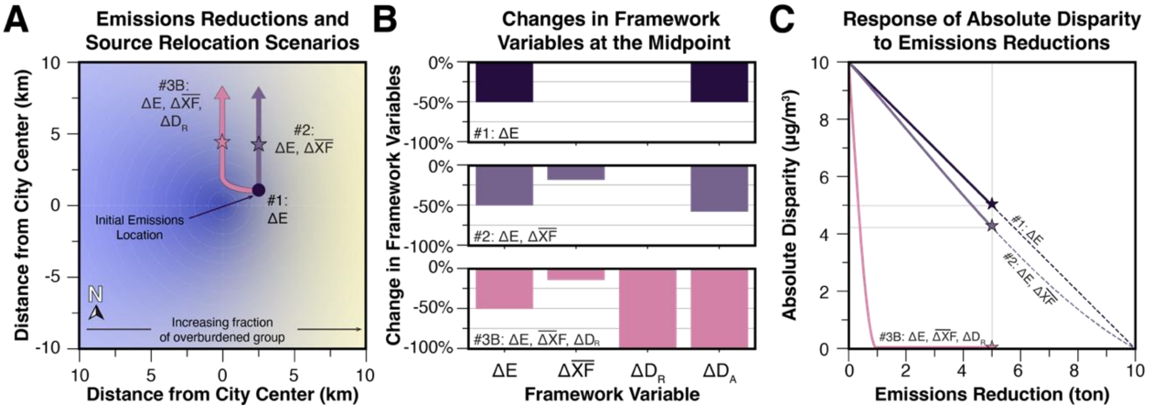

Fig. S3. Absolute disparity is mitigated very expeditiously with aggressive reductions in relative disparity. The first two scenarios from Fig. 2 are compared with new Policy #3B, in which the emission location is aggressively shifted towards the center of the population distribution. (**A**) The three theoretical emission reduction scenarios are depicted atop a simplified city. In Scenario #1, emissions are reduced in-place; in Scenarios #2 and #3B, the average source location moves along the arrow as emissions are reduced. The stars represent the point at which the absolute disparity has reduced by approximately 50% in each curve; the influence of changes from each framework variable at this point are depicted in (**B**). (**C**) The absolute disparity in exposure for members of the overburdened group is calculated for each policy at each model step. Unlike in Fig. 2, here, Scenario #3B results in zero absolute disparity by the time emissions are reduced by 50%.

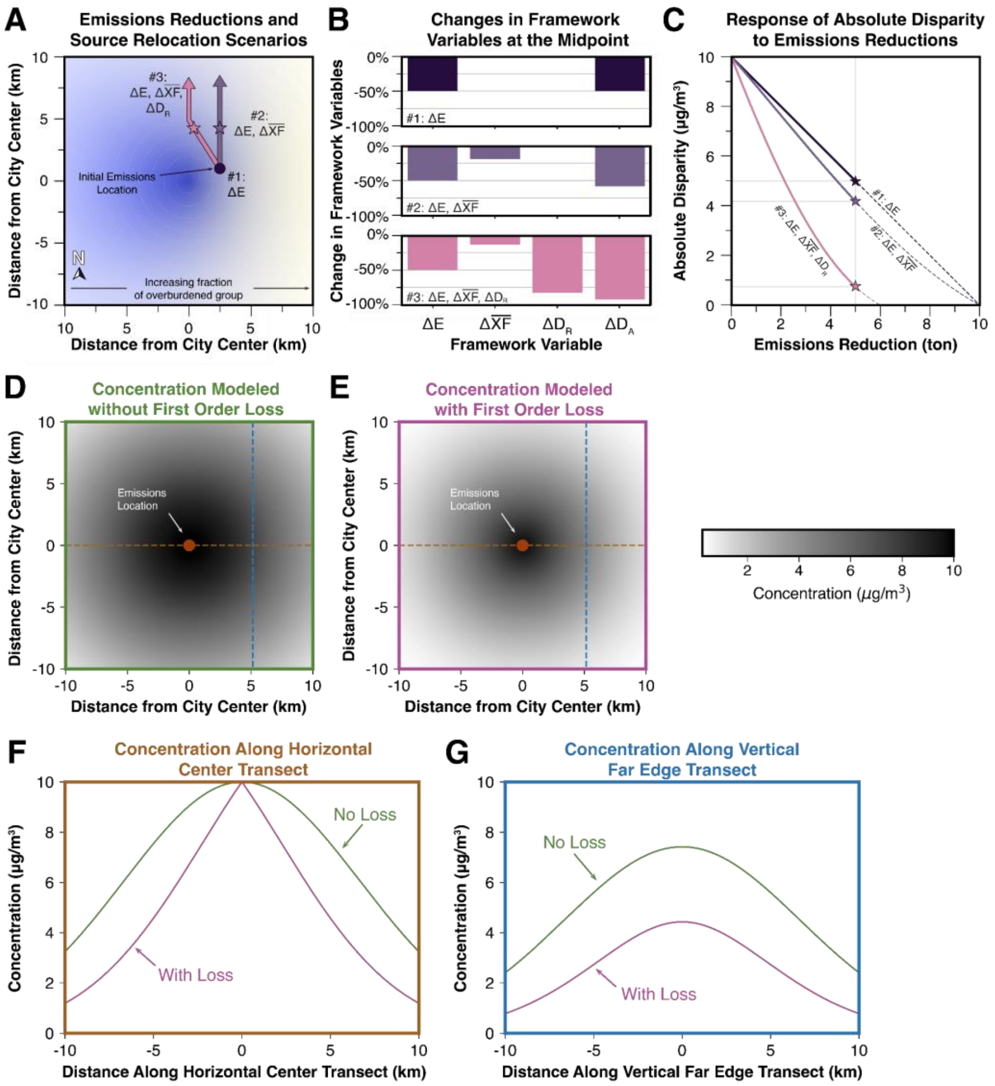

Fig. S4. Adding first order rate loss to the illustrative model does not change the key insight of Fig. 2. The same three scenarios modeled in Fig. 2 are repeated using a slightly more complex underlying illustrative model. Here, a relatively rapid first order loss process (larger than would be expected for deposition or many chemical transformations) is added to the concentration estimated across space at each scenario step of the model. While the underlying concentration pattern changes, the insights from the framework do not. (**A**) The emission reduction pathways and population distribution are unchanged. The resulting changes in framework variables at both (**B**) the 50% emission reduction and the (**C**) along the full emission mitigation pathway are virtually unchanged. While still nearly negligible, Scenario #2 resulted in the largest change from Fig. 2, likely due to differences in the steepness of the concentration distribution relative to the population distribution. The concentration for a single point release at the center is illustrated (**D**) without first order loss and (**E**) with first order loss. As expected, the first order loss function results in a steeper gradient of concentrations from the release point. Two transects are drawn in panels (**D**) and (**E**); the concentrations along these transects are shown in (**F**) and (**G**).

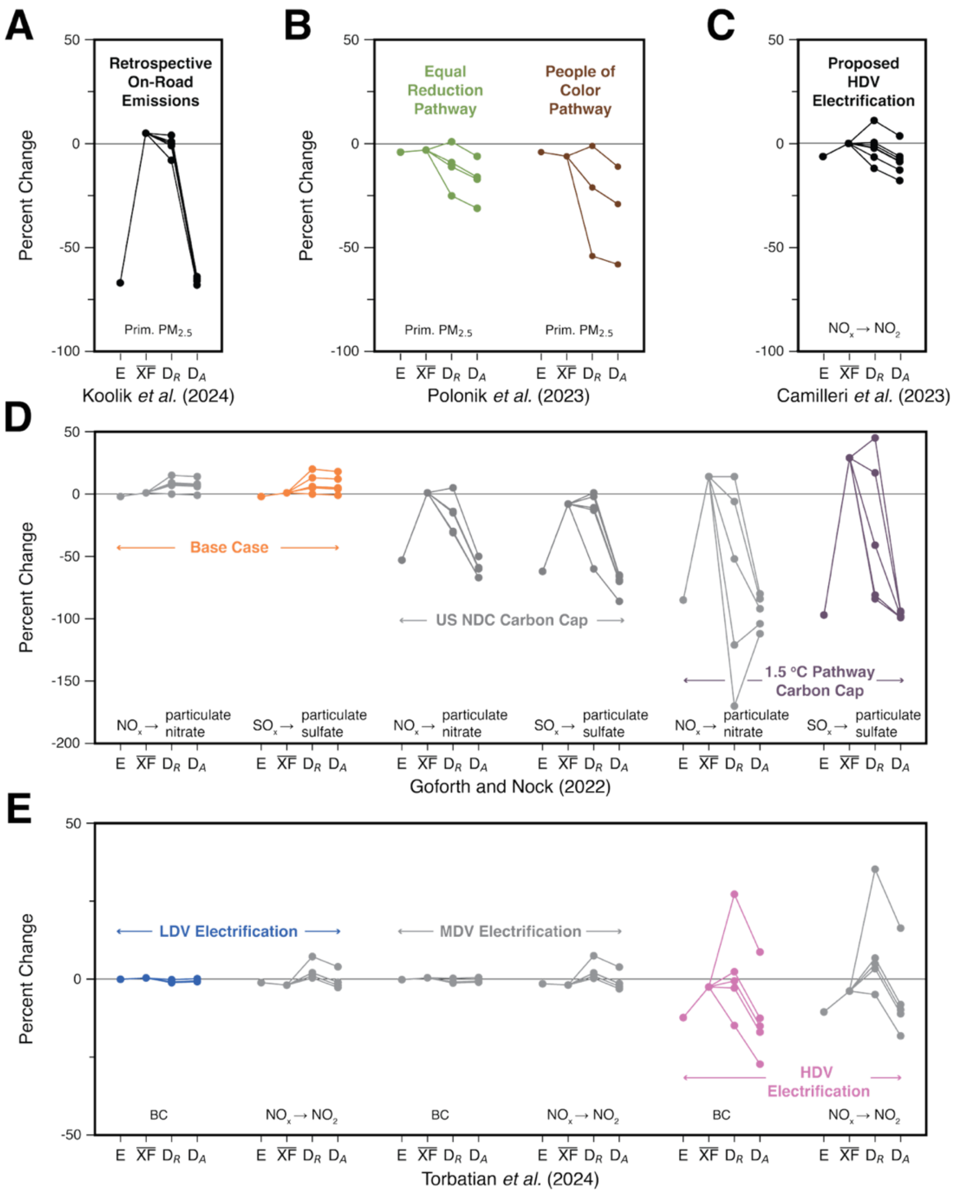

Fig. S5. Additional policies and pollutants from the literature are evaluated using the framework variables. Whereas in Fig. 3 we show only the groups with the minimum and maximum change in D_A, here we demonstrate the impacts on all demographic groups included in each study. (**A**) through (**C**) are the same scenarios included in Fig. 3. (**D**) and (**E**) include an additional scenario and an additional pollutant for reference. All policies are labelled with their policy titles from the original analysis and the pollutant of interest. Results from Goforth and Nock (2022) and Torbatian et al. (2024) demonstrate how the choice of pollutant of interest does not dramatically affect the insight that the framework provides about the underlying mechanism driving reductions in disparity. Where a pollutant label includes an arrow, chemistry in the underlying model has transformed the pollutant emitted (left side of arrow) into the concentration of the pollutant of interest (right side of arrow). Black or colored lines indicate curves drawn as part of Fig. 3; the gray lines are additional policy scenarios or pollutants. Additional details about the methods employed and policies evaluated in each of these analyses, as well as commentary about the major insights derived from the application of our conceptual framework, are included in Table S1.

Table S1. Application of the conceptual framework to a previously peer-reviewed studies in the literature.
Here, we estimate the percent change in each framework variable for each demographic group across various policies. For ease of interpretation, positive changes (increases across time) are shaded in light red, negative changes (decreases across time) are shaded in light green, and nearly unchanged (+/- 2%) are shaded in gray. A subset of these results are shown in Fig. 3 and all are shown in Fig. S5; these are denoted in the "Fig." column. A unifying theme of this analysis is that policies intended to mitigate exposure disparities are more effective at reducing relative and absolute disparities.

Ref.	Method	Policy Evaluated	Poll.	Fig.	Group	Percent Change in Framework Variable				Commentary
						ΔE	ΔXF	ΔD_R	ΔD_A	
[5]	InMAP modeling of NOx and SOx concentrations associated with emissions changes from decarbonization pathways estimated by the Regional Energy Deployment System (ReEDS) from the National Renewable Energy Lab (NREL). Here, we take a snapshot of these changes between 2020 and 2030.	**Base Case**: a business-as-usual scenario assuming no new carbon policies are implemented	NO_X	Fig. S5D	Asian			15%	14%	In the Base Case, the lack of decarbonization effort has led to a relatively low decrease in emissions, and an increase in disparity. Because of the modest changes in emissions and average exposure factor, the increases in relative disparity directly translate to changes in absolute disparity. With the NDC Carbon Cap Scenario, a more substantial reduction in emissions occurs with a small change in the average exposure factor. The bulk of the reduction in absolute exposure disparity is likely due to the large reductions in emissions (53% and 62% reduction), but the variation in the change in absolute disparity across groups is directly tied to the change in relative disparity by group. Compare, for example, the change in absolute disparity in exposure to NO_x for Black versus Latinx/Hispanic people. Both groups experience a substantial reduction in absolute disparity, however the absolute disparity in exposure to Latinx/Hispanic people is much larger (67% reduction versus 50% reduction) due to the large reduction in relative disparity (31% reduction versus 5% increase). In the 1.5 °C Pathway Scenario, the average exposure factor increases for all people, but this increase is counteracted by substantial reductions in emissions and relative disparity. The absolute disparity in exposure to SO_x for all groups is nearly ameliorated (in large part due to the near-elimination of SO_x emissions). The absolute disparity in exposure to NO_x is mitigated for Latinx/Hispanic and Asian people, while more work is needed to reduce the absolute disparity for Black people.
					Black			7%	6%	
					Indigenous	-2%	1%	8%	7%	
					Latinx/Hispanic			9%	8%	
					Non-Latinx White			0%	-1%	
			SO_X	Fig. 3G, Fig. S5D	Asian			5%	4%	
					Black			13%	12%	
					Indigenous	-2%	1%	20%	18%	
					Latinx/Hispanic			6%	5%	
					Non-Latinx White			0%	-1%	
		**US NDC Carbon Cap**: scenario assumes the United States implements a carbon cap, per the Nationally Determined Contributions determined in the 2015 Paris Agreement	NO_X	Fig. S5D	Asian			-30%	-67%	
					Black			5%	-50%	
					Indigenous	-53%	1%	-15%	-60%	
					Latinx/Hispanic			-31%	-67%	
					Non-Latinx White			-14%	-59%	
			SO_X	Fig. S5D	Asian			1%	-65%	
					Black			-13%	-70%	
					Indigenous	-62%	-8%	-60%	-86%	
					Latinx/Hispanic			-11%	-69%	
					Non-Latinx White			-2%	-66%	
		**1.5 °C Pathway Carbon Cap**: policy scenario required to reduce the net global warming to 1.5 °C	NO_X	Fig. S5D	Asian			-170%	-112%	
					Black			14%	-80%	
					Indigenous	-85%	14%	-6%	-84%	
					Latinx/Hispanic			-121%	-104%	
					Non-Latinx White			-52%	-92%	
			SO_X	Fig. 3H, Fig. S5D	Asian			-84%	-99%	
					Black			45%	-94%	
					Indigenous	-97%	29%	17%	-95%	
					Latinx/Hispanic			-81%	-99%	
					Non-Latinx White			-41%	-98%	

Table S1, continued

Ref.	Method	Policy Evaluated	Poll.	Fig.	Group	Percent Change in Framework Variable				Commentary
						ΔE	Δ*XF*	ΔD_R	ΔD_A	
[6]	InMAP modeling of primary $PM_{2.5}$ concentrations from idealized emission reduction pathways, bias corrected at the tract-level using mean surface $PM_{2.5}$ observational data.	**Equal Reduction Pathway**: greenhouse gas and co-pollutant emissions are reduced by the same proportion everywhere.	Prim. $PM_{2.5}$	Fig. 3F, Fig. S5B	Asian	-4%	-3%	-11%	-17%	Both pathways result in approximately the same emission reduction, compared to the unmodified emissions scenario, but the changes in exposure disparity vary. The People of Color pathway is a great example of the type of policy design that prioritizes reductions in relative disparity. This is demonstrated in the resulting change in absolute disparity: the change in absolute disparity in the People of Color pathway outpaces that of the Equal Reduction pathway for all groups, resulting in a more accelerated reduction in absolute disparity for the same change in emissions.
					Black			1%	-6%	
					Latino			-25%	-31%	
					People of Color			-9%	-16%	
					White			-9%	-16%	
		**People of Color Pathway**: greenhouse gas and co-pollutant emission reductions are prioritized in areas with the highest fraction of people of color.		Fig. 3E, Fig. S5B	Asian	-4%	-6%	-21%	-29%	
					Black			-1%	-11%	
					Latino			-54%	-58%	
					People of Color			-21%	-29%	
					White			-21%	-29%	
[7]	InMAP Source-Receptor Matrix modeling of primary $PM_{2.5}$ concentrations from regulatory model of on-road emissions (EMFAC2021)	**Aggregate Mobile Source Policy**: On-road mobile emissions reductions in California from 2000 to 2019.	Prim. $PM_{2.5}$	Fig. 3A, Fig. S5A	Asian	-67%	5%	1%	-65%	The reduction in absolute disparity in exposure is most directly tied to a system-wide change in emissions, and not the spatial distribution of those emissions. As a result, the absolute disparity in exposure changes nearly uniformly across groups and at approximately the same rate as the emissions.
					Black			-8%	-68%	
					Hispanic			0%	-66%	
					White			-1%	-66%	
					Other			4%	-64%	
[8]	Two-way coupled CMAQ (v5.2) and WRF (v3.8) simulations of NO_x concentrations using emissions from SMOKE and MOVES.	**Truck Electrification**: The instantaneous transition of 30% of internal combustion engine heavy duty trucks to electric in the Greater Chicago area.	NO_X	Fig. 3B, Fig. S5C	Asian	-7%	-0.2%	-7%	-13%	The change in average exposure factor is nearly zero, so the combined effects of changes in emissions and relative disparity are directly demonstrated in the changes in absolute disparity. The spatial patterns of heavy-duty truck electrification lead to larger reductions in relative disparity for Asian and Native American people, yielding a higher rate of change in absolute disparity. For groups with minimal change in relative disparity (e.g., Black, Hispanic/Latino, Other, White), the reduction in absolute disparity is effectively just from the reduction in system-wide emissions.
					Black			-2%	-9%	
					Hispanic/Latino			-2%	-9%	
					Native American / Alaskan Native			-12%	-18%	
					Native Hawaiian / Pacific Islander			11%	3%	
					Other			0%	-6%	
					White			-1%	-8%	

Table S1, continued

Ref.	Method	Policy Evaluated	Poll.	Fig.	Group	Percent Change in Framework Variable				Commentary
						ΔE	Δ*XF*	ΔD_R	ΔD_A	
[9]	Chemical-transport modeling (Polair3D) of BC and NO_2 concentrations resulting from modeled changes in vehicle electrification in the Greater Toronto area relative to a base case scenario estimated from national emission inventories (Canada's Air Pollutant Emission Inventory and the US's National Emissions Inventory).	**LDT Electrification:** full electrification of all light-duty trucks in the Greater Toronto area. These trucks primarily circulate on local roads and neighorhoods.	BC	Fig. S5E	Deprivation Q1	-0.1%	0.4%	-1%	-1%	The three vehicle electrification scenarios result in different equity outcomes. HDV electrification has the largest reduction in absolute disparity. This reduction in absolute disparity arises in large part because of the large reduction in emissions, but also due to a slightly larger reduction in exposure factor. Interestingly, relative disparity in exposure to NO_2 increases for the higher deprivation quintiles for all three scenarios. The direct result in this is a smaller change in absolute disparity for these higher quintiles. The third deprivation quintile increases in disparity in all cases, likely due to the position of these population clusters relative to roadway and highways. There are minimal differences between the spatial distribution or emission reductions for the LDT and MDT electrification scenarios.
					Deprivation Q2			-1%	-1%	
					Deprivation Q3			0%	0%	
					Deprivation Q4			-1%	-1%	
					Deprivation Q5			-1%	-1%	
			NO_2	Fig. S5E	Deprivation Q1	-1%	-2%	0%	-3%	
					Deprivation Q2			2%	-1%	
					Deprivation Q3			7%	4%	
					Deprivation Q4			1%	-2%	
					Deprivation Q5			2%	-1%	
		**MDT Electrification:** full electrification of all medium-duty trucks in the Greater Toronto area. These trucks primarily circulate on highways and major roads.	BC	Fig. 3C, Fig. S5E	Deprivation Q1	-0.2%	0.4%	-1%	-1%	
					Deprivation Q2			-1%	-1%	
					Deprivation Q3			0%	1%	
					Deprivation Q4			-1%	-1%	
					Deprivation Q5			-1%	-1%	
			NO_2	Fig. S5E	Deprivation Q1	-1%	-2%	0%	-3%	
					Deprivation Q2			2%	-1%	
					Deprivation Q3			7%	4%	
					Deprivation Q4			1%	-2%	
					Deprivation Q5			2%	-1%	
		**HDT Electrification:** full electrification of all heavy-duty trucks in the Greater Toronto area. These trucks primarily circulate on highways.	BC	Fig. 3D, Fig. S5E	Deprivation Q1	-12%	-3%	-15%	-27%	
					Deprivation Q2			2%	-13%	
					Deprivation Q3			27%	9%	
					Deprivation Q4			-3%	-17%	
					Deprivation Q5			-1%	-15%	
			NO_2	Fig. S5E	Deprivation Q1	-11%	-4%	-5%	-18%	
					Deprivation Q2			7%	-8%	
					Deprivation Q3			35%	16%	
					Deprivation Q4			3%	-11%	
					Deprivation Q5			5%	-10%	

SI References

1. D. H. Bennett, et al., Defining intake fraction. *Environ. Sci. Technol.* **36**, 206A–211A (2002).
2. J. D. Marshall, W. J. Riley, T. E. McKone, W. W. Nazaroff, Intake fraction of primary pollutants: motor vehicle emissions in the South Coast Air Basin. *Atmos. Environ*. **37**, 3455–3468 (2003).
3. J. D. Marshall, S.-K. Teoh, W. W. Nazaroff, Intake fraction of nonreactive vehicle emissions in US urban areas. *Atmos. Environ*. **39**, 1363–1371 (2005).
4. J. S. Apte, E. Bombrun, J. D. Marshall, W. W. Nazaroff, Global intraurban intake fractions for primary air pollutants from vehicles and other distributed sources. *Environ. Sci. Technol*. **46**, 3415–3423 (2012).
5. T. Goforth, D. Nock, Air pollution disparities and equality assessments of US national decarbonization strategies. *Nat. Commun.* **13**, 7488 (2022).
6. P. Polonik, K. Ricke, S. Reese, J. Burney, Air quality equity in US climate policy. *Proc. Natl. Acad. Sci. U.S.A.* **120**, e2217124120 (2023).
7. L. H. Koolik, *et al.*, $PM_{2.5}$ exposure disparities persist despite strict vehicle emissions controls in California. *Sci. Adv.* **10**, eadn8544 (2024).
8. S. F. Camilleri, *et al.*, Air quality, health and equity implications of electrifying heavy-duty vehicles. *Nat. Sustain.* **6**, 1643–1653 (2023).
9. S. Torbatian, *et al.*, Societal co-benefits of zero-emission vehicles in the freight industry. *Environ. Sci. Technol*. **58**, 7814–7825 (2024).
